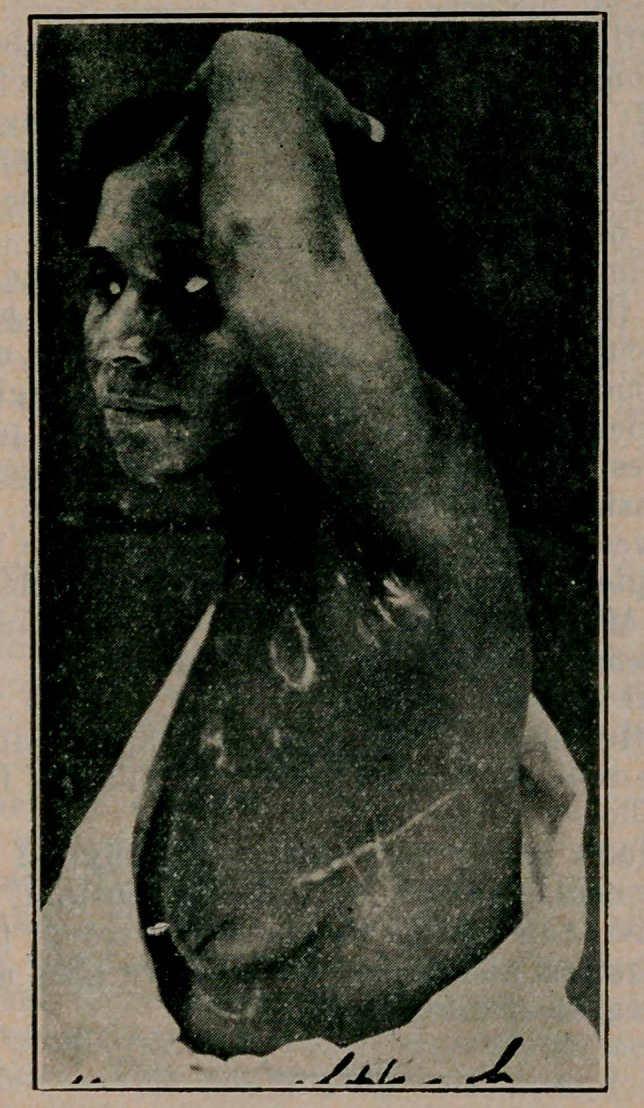# Incised Wound of Heart, Suture and Recovery

**Published:** 1916-04

**Authors:** 


**Incised Wound of Heart, Suture and Recovery.** Dr. Wm. J. Coleman, Baltimore, Maryland Medical Journal, Feb. 1916. (Cut by courtesy of editor). A colored woman, aged 36, had been slashed across the chest with a razor. Two wounds had been made, one superficial in the left breast (showing as pigmented line outside of nipple the other much longer and deeper, penetrating the thoracic wall, with collapse of lung, and opening the pericardium for a length of 2 c.m. 10 c.m. of the fifth rib was resected, after controling superficial haemorrhage and the pericardial wound was enlarged to expose a wound in the left ventricle. Three catgut interrupted sutures were placed in the heart, a cigarette drain introduced into the pericardium and the pericardium sutured down to the drain. The left pleural cavity was cleaned by sponging, two cigarette drains placed and the muscles and integument closed with catgut and silkworm gut. Recovery was uneventful. Simon collected up to 1912, 241 operations for gunshot wounds of the heart with 124 deaths (51%) and 200 for stab wounds with 99 deaths (49%).

## Figures and Tables

**Figure f1:**